# In-Person Interpreter Use and Hospital Length of Stay among Infants with Low Birth Weight

**DOI:** 10.3390/ijerph15081570

**Published:** 2018-07-25

**Authors:** Monica Eneriz-Wiemer, Lee M. Sanders, Mary McIntyre, Fernando S. Mendoza, D. Phuong Do, C. Jason Wang

**Affiliations:** 1 Department of Pediatrics, Palo Alto Medical Foundation, Los Gatos, CA 95032, USA; 2 Division of General Pediatrics, Stanford University School of Medicine, Stanford, CA 94305, USA; leesanders@stanford.edu (L.M.S.); fernando.mendoza@stanford.edu (F.S.M.); cjwang1@stanford.edu (C.J.W.); 3 Center for Policy, Outcomes, and Prevention, Stanford University, Stanford, CA 94305, USA; 4 Lucile Packard Children’s Hospital at Stanford, Palo Alto, CA 94304, USA; MMcIntyre@stanfordchildrens.org; 5 Zilber School of Public Health, University of Wisconsin-Milwaukee, Milwaukee, WI 53201, USA; dphuong@uwm.edu

**Keywords:** children’s health, language, healthcare disparities, length of stay, health services research

## Abstract

To ensure timely appropriate care for low-birth-weight (LBW) infants, healthcare providers must communicate effectively with parents, even when language barriers exist. We sought to evaluate whether non-English primary language (NEPL) and professional in-person interpreter use were associated with differential hospital length of stay for LBW infants, who may incur high healthcare costs. We analyzed data for 2047 infants born between 1 January 2008 and 30 April 2013 with weight <2500 g at one hospital with high NEPL prevalence. We evaluated relationships of NEPL and in-person interpreter use on length of stay, adjusting for medical severity. Overall, 396 (19%) had NEPL parents. Fifty-three percent of NEPL parents had documented interpreter use. Length of stay ranged from 1 to 195 days (median 11). Infants of NEPL parents with no interpreter use had a 49% shorter length of stay (adjusted incidence rate ratio (IRR) 0.51, 95% confidence interval (CI) 0.43–0.61) compared to English-speakers. Infants of parents with NEPL and low interpreter use (<25% of hospital days) had a 26% longer length of stay (adjusted IRR 1.26, 95% CI 1.06–1.51). NEPL and high interpreter use (>25% of hospital days) showed a trend for an even longer length of stay. Unmeasured clinical and social/cultural factors may contribute to differences in length of stay.

## 1. Introduction

Preterm birth and low birth weight (LBW) are common causes of mortality and morbidity worldwide [1]. These conditions also create a significant socioeconomic burden. In the United States alone, these conditions account for half of infant hospitalization costs and one-quarter of pediatric hospitalization costs at an estimated $5.8 billion per year [2]. To improve health outcomes and reduce costs, attention has focused on prevention of preterm/LBW births. Yet, for those parents who deliver preterm/LBW infants, there is a great need to understand how best to prepare parents for infants’ discharge from the neonatal intensive care unit (NICU) [3]. Parents must learn how to manage infants’ medical conditions, including knowing when and how to respond to changes in their infants’ clinical status and where to obtain follow-up care [3]. Fundamentally, this preparation requires communication and coordination between parents and healthcare providers to ensure timely hospital discharge and optimal health outcomes [3]. 

However, communication is difficult when parents and healthcare providers do not speak the same language. In the United States, where 15% of infants are born to one or more parents who speak a non-English primary language (NEPL), language barriers impact many families’ experiences with healthcare. Parents with NEPL report poor communication with healthcare providers [4,5,6] and lower satisfaction with their children’s healthcare [4]. Children of parents with NEPL experience fewer outpatient referrals [4,7] and delayed hospital discharge [7]. While US law and national guidelines mandate the use of interpreter services in all healthcare organizations [8], interpreters are underused by healthcare providers even when they are readily available [9,10,11,12,13,14,15]. The extent to which professional in-person interpreters are used in NICUs is unknown and likely highly variable across institutions, which may impact the quality of care for infants treated in NICUs. 

Hospital length of stay is associated with the quality, efficiency, and cost of care [16]. It is known that lengths of stay that are too short [17] or too long [18] may be inappropriate. From an ethical standpoint, appropriateness of care leading to timing of patient discharge should be determined by the patient’s health status and should not be impacted by the parents’ and/or patients’ NEPL status or ability to pay. Therefore, understanding the relationship between language and professional in-person interpreter use on hospital length of stay may provide an important basis for improving quality of healthcare and reducing costs associated with preterm/LBW birth. In this study, we sought to understand whether parents’ NEPL was associated with differential hospital length of stay for LBW infants born and discharged from one academic medical center with a high prevalence of NEPL. Given the increased communication burden between healthcare providers and NEPL families, we hypothesized that NEPL would be associated with longer hospital length of stay and that use of professional in-person interpreters would moderate this association.

## 2. Materials and Methods

We examined comprehensive clinical and administrative data on all LBW infants born between 1 January 2008 and 30 April 2013 at Lucile Packard Children’s Hospital at Stanford (LPCH), a regional referral center for high-risk obstetrics and neonatal care. This hospital serves ethnically and linguistically diverse families: half of the residents in the surrounding areas (Santa Clara and San Mateo counties) report speaking non-English languages at home [19]. Professional in-person interpreters are available in Spanish 24 hours per day, 7 days per week and in Cantonese, Mandarin, Vietnamese, Russian, and American Sign Language during weekday business hours. Telephonic interpretation in over 200 spoken languages is used when in-person interpreters are unavailable. 

Infants were included if they had low birth weight (LBW), defined as <2500 g. Infants were excluded if they were (a) born with gestational age <22 weeks (less than typical threshold of viability); (b) transferred into or out of LPCH; (c) died prior to hospital discharge; or (d) if parent primary language was unknown. Parents reported their preferred primary language to hospital representatives at time of patient registration. 

We extracted detailed clinical, sociodemographic, and administrative data from the electronic health record for each infant’s birth hospitalization (beginning on date of birth and ending on date of hospital discharge) using a standards-based informatics platform called the Stanford Translational Research Integrated Database Environment (STRIDE) [20]. Four distinct datasets were extracted containing information on (a) clinical and sociodemographic characteristics, including parent primary language; (b) surgeries received; (c) diagnostic billing (ICD-9) codes; and (d) clinical encounter logs recording individual encounters with professional in-person interpreters. Datasets was sequentially cleaned and merged using a common linker (encounter identification numbers) unique to each birth hospitalization. Analyses were conducted using Stata 12 software (StataCorp. 2011. Stata Statistical Software: Release 12. College Station, TX, USA: StataCorp LP). We obtained approval from Stanford University Institutional Review Board prior to study activities.

Two key independent variables were assessed. First, infants of parents with NEPL were compared to those of English-speaking parents. To account for effect of in-person interpreter use, an adjusted rate of interpreter use was calculated consisting of the total number of in-person interpreter encounters divided by the infant’s expected duration of hospital stay as a function of gestational age (term gestation of 294 days minus the infant’s gestational age in days). We used a term gestation of 42 weeks (294 days) as the longest predicted length of stay so that all denominators would be non-zero. When the resulting rates of in-person interpreter use were plotted for all infants of parents with NEPL, we determined that the top 5% of parents using in-person interpreters used interpreters for 25% or more of their infant’s total days. We then created a categorical variable accounting for NEPL plus the level of in-person interpreter use: (a) high in-person interpreter use (>25% of days); (b) low in-person interpreter use (1–24% of days); and (c) no in-person interpreter use.

Analyses were also adjusted for clinical and sociodemographic covariates. Clinical covariates included male gender (reference: female), extreme low birth weight <1000 g (reference: 1001–2500 g), and neonatal medical index scores for severity of disease. The neonatal medical index is a validated clinical severity scoring system ranging from I (least severe disease) to V (most severe disease) that predicts the cognitive and motor development of preterm/LBW infants in the first three years of life [21,22]. Clinical factors used in calculating neonatal medical index scores include birth weight, total days on assisted ventilation, major surgeries, and complications such as intraventricular hemorrhage (Appendix Table A1). Infants with neonatal medical index scores of IV–V were grouped and compared to infants with scores of I–III. Sociodemographic covariates included public insurance (reference: private insurance), maternal married status (reference: single, separated, or divorced), and black race (reference: non-black). Non-black race included White, Asian, Native American, Native Hawaiian/Pacific Islander, and other. Parents reported their race and ethnicity to hospital representatives at the time of patient registration.

We conducted two-tailed chi-squared tests to compare differences in clinical and sociodemographic characteristics between infants of parents with NEPL versus infants of English-speakers. Since our length of stay distribution demonstrated overdispersion, we completed multivariate analyses using zero-truncated negative binomial regressions. We transformed our beta coefficients into incidence rate ratios (IRRs). In the first regression model, we included our first key independent variable of NEPL. In the second regression model, we included our categorical variable for NEPL plus the level of in-person interpreter use. Both models were adjusted for all clinical and sociodemographic covariates.

## 3. Results

Data were obtained for 2871 infants born in the study period with birth weight <2500 g. Of these, 824 were excluded for one or more of the following reasons: 25 with gestational age <22 weeks, 691 transfers, 131 died prior to hospital discharge, and 8 with missing parent language. Of the 2047 infants in our final sample, 396 (19%) had parents with NEPL. Among them, 360 (91%) parents with NEPL reported Spanish language and 36 (9%) reported 1 of 20 other languages. Infants of parents with NEPL were similar to infants of English-speakers except that infants of parents with NEPL were much more likely to be Hispanic white (*p*-value 0.000) and publicly insured (*p*-value 0.000) compared to infants of English-speakers (Table 1). Parents with NEPL were also less likely to be married compared to English-speakers (*p*-value 0.000). Among infants of parents with NEPL, 185 (47%) had no in-person interpreter use, 188 (47%) had low in-person interpreter use (1–24% of days), and 23 (6%) had high in-person interpreter use (>25% of days). Among infants whose parents reported English primary language, 53 (3%) parents had at least one in-person interpreter encounter during the birth hospitalization. 

Length of stay for the birth hospitalization was a right-skewed distribution ranging from 1 to 195 days with a median of 11 days (standard deviation 28 days, interquartile range of 26 days). In model 1 (NEPL as key independent variable), infants of parents with NEPL demonstrated the same hospital length of stay (IRR 0.89, 95% confidence interval 0.77–1.03, *p*-value 0.118) compared to infants of English-speakers (Table 2). In model 2 (including NEPL plus level of in-person interpreter use as independent variables), NEPL and no in-person interpreter use was associated with a 49% shorter length of stay (IRR 0.51, 95% confidence interval 0.43–0.61, *p*-value 0.000; mean 7.0 days shorter with 95% confidence interval 5.5–8.1 days) compared to English-speakers. NEPL and low in-person interpreter use was associated with a 26% longer length of stay (IRR 1.26, 95% confidence interval 1.06–1.51, *p*-value 0.010; mean 3.7 days longer with 95% confidence interval 0.9–7.2 days) compared to English-speakers. NEPL and high in-person interpreter use showed a trend for an even longer length of stay although this association was not significant (IRR 1.41, 95% confidence interval 0.89–2.22, *p*-value 0.145). Male gender, extreme low birth weight (<1000 g), and severe medical condition were associated with longer lengths of stay in both models compared to reference groups. There were no associations between sociodemographic variables and length of stay, except in model 2 in which maternal married status was associated with a 14% shorter length of stay (IRR 0.86, 95% confidence interval 0.76–0.97, *p*-value 0.017) compared to single, separated, or divorced status.

## 4. Discussion

In our study of LBW infants in a large regional children’s hospital where one-fifth of parents speak a NEPL, we found that when examined as a whole, infants of parents with NEPL demonstrated no difference in length of stay compared to infants of English-speakers. However, we found that only half of our NEPL subjects received professional in-person interpreter services. When we compared NEPL parents with in-person interpreter use to NEPL parents without in-person interpreter services, we found that the level of in-person interpreter use among parents with NEPL was an independent predictor of increased length of stay for LBW infants. Infants of NEPL parents with no in-person interpreter use had a shorter length of stay (mean 7.0 days shorter) compared to English-speakers. In contrast, infants of NEPL parents with in-person interpreter use had a longer length of stay (mean 3.7 days longer) compared to English-speakers. 

This finding may reflect a bias among healthcare providers to use professional in-person interpreters only for patients with greater clinical, social, and/or cultural complexity. Prior studies show that providers may use their own limited foreign-language skills or ad hoc interpreters for less clinically complex patients but opt for professional in-person interpreters for more complex patients [10,11]. This was shown to be true in a similar study of hospitalized adults: NEPL patients with no in-person interpreter use had a shorter length of stay and NEPL patients with in-person interpreter use had a longer length of stay than English-speakers [23]. The authors concluded that increased clinical complexity, rather than in-person interpreter use, likely accounted for longer length of stay in these patients [23]. However, in our study, measures for clinical complexity were taken into account in all regressions and use of in-person interpreters continued to have an effect on length of stay. Healthcare providers may also be more likely to use in-person interpreters when interacting with parents with differing social/cultural beliefs. In a qualitative study of NICU providers caring for infants of immigrants, providers were more likely to use in-person interpreters in times of infant health crisis and at hospital discharge, as they perceived that parents’ cultural, ethnic, and religious beliefs and practices added complexity and potential misunderstanding to healthcare encounters [24]. When clinical, social, or cultural complexity are present and in-person interpreters are used to enhance communication between parents and healthcare providers, these encounters likely represent a higher quality of healthcare. 

Since the family backgrounds of our subjects may be quite varied, unmeasured clinical, social, or cultural factors may play important roles in interpreting these findings. For example, shorter length of stay may indicate that infants of parents with NEPL are inherently healthier as parents with NEPL in our study were primarily Hispanic immigrants. Prior studies examining the so-called “immigrant paradox” suggest that infants of Hispanic immigrants may be healthier than infants of US-born Hispanic parents due to unmeasured, culturally-embedded health behaviors such as reduced rates of maternal smoking, alcohol use, and obesity [25,26]. Social and cultural factors may also facilitate earlier discharge. Social networks in immigrant communities may provide essential emotional and tangible support enabling parents to feel comfortable with earlier hospital discharge [26,27]. Yet, these all depend on understanding the family’s circumstances through effective communication. Another issue may be that parents with NEPL are disinclined to ask for interpreters [28] and less likely to ask questions and participate in medical decision-making compared to English-speakers [29]. Normative cultural values such as simpatia (politeness and avoidance of confrontation) and respeto (importance of showing respect to authority figures) may contribute to lack of self-advocacy if parents perceive such behaviors may create conflict or show disrespect [27]. 

This study has several limitations. This retrospective analysis represents the experience of one institution and may not generalize to other institutions. However, since the study hospital is in a major metropolitan area where up to half of families speak non-English languages, we believe our findings provide important insights for institutions in similar areas that provide neonatal care to infants from non-English-speaking, immigrant families. Our indicator for NEPL may also be subject to measurement bias, as we used parent self-reported primary language, which may be miscoded. Parents’ actual level of English proficiency was not captured during infants’ hospitalizations. Level of English proficiency may have been discordant between infants’ parents; for example, one parent may have been English proficient while the other may have had limited English proficiency. In families with one English proficient parent, parents may not have been offered professional interpreters during health care encounters. Our measure of in-person interpreter use also may have underestimated the actual use of interpreter services: we captured only professional in-person interpreter encounters but did not capture encounters with telephonic interpreters or bilingual providers. By contrast, telephonic interpreters and bilingual providers are common at this and other institutions, and their use may facilitate timely and appropriate discharge of NEPL families. Further study is needed to measure use of telephonic interpreters and bilingual providers in NICU settings and understand their relative contributions to provision of care for NEPL families. 

Despite these limitations, our study presents the first analysis of the relationships between language, in-person interpreter use, and hospital length of stay for LBW infants, and it may carry important implications for the healthcare provided to infants and children from NEPL communities. While a shorter hospital length of stay may be appropriate, we do not know actual implications on quality of care for infants of parents with NEPL. One overarching concern is low use of professional in-person interpreters in our sample, with only half of parents with NEPL receiving in-person interpreters. Although Title VI of the 1964 Civil Rights Acts mandates that all US healthcare organizations receiving federal funds provide access to interpreter services, and use of interpreter services is a key component of national guidelines for provision of culturally and linguistically appropriate healthcare, the type of interpreter services under various conditions is not specified [8]. The acuity and complexity of neonatal intensive care would suggest that providing services with in-person interpreters at least once during the hospitalization would be appropriate. Moreover, increased use of in-person interpreters within a family-centered model of neonatal intensive care is likely to be cost-effective [8,30]. For example, specific use of in-person interpreters during transitions (admission and discharge) have been shown to reduce hospital length of stay for adult patients with NEPL [31]. Given the diversity of languages spoken by US parents with NEPL, the use of professional telephonic interpretation should be made available where in-person interpreters are not feasible [28,32]. Future study should examine how and when all modes of interpretation services are used in NICU and other ICU settings as well as how interpreter use may affect outcomes following discharge, such as readmissions and unmet health needs [33]. Furthermore, observational studies are needed to assess what information is being communicated to parents in NICUs and to determine which educational methods and/or modalities may be most effective for teaching parents important health information, regardless of primary language [34]. Population-level data, including clinic visits and hospital readmissions across institutions, may help answer these critical questions.

## 5. Conclusions

As worldwide migration patterns increasingly bring healthcare providers in contact with diverse populations, healthcare that bridges language and social/cultural differences is crucial. Our study suggests that in-person interpreters can affect length of stay, suggesting improved communication and possibly improved care. Further research into parent language and interpretation services may provide opportunities to improve quality and reduce costs of healthcare for children with LBW, who remain vulnerable for health and developmental problems long after hospital discharge. 

## Figures and Tables

**Table 1:** Characteristics of the final sample of infants with low birth weight with respect to parents’ primary language status (*N* = 2047).

Characteristic	Non-English Primary Language * (*N* = 396)	English Primary Language (*N* = 1651)
Male Gender	190 (48%)	760 (46%)
Extreme Low Birth Weight (<1000 g)	26 (7%)	103 (6%)
Severe Medical Condition (NMI ** score IV or V)	58 (15%)	198 (12%)
Hispanic White Race ^†^	343 (87%)	252 (15%)
Black Race ^†‡^	0 (0%)	92 (6%)
Public Insurance ^†^	355 (90%)	438 (27%)
Married ^†^	192 (51%)	1227 (76%)

* Among parents with non-English primary language, 185 (47%) had no professional in-person interpreter use, 188 (47%) had low in-person interpreter use (1–24% of infant’s hospital days), and 23 (6%) had high in-person interpreter use (>25% of infant’s hospital days); ** Abbreviation: NMI = neonatal medical index; ^†^ *p*-value 0.000; ^‡^ Black race included those with both non-Hispanic (89) and Hispanic ethnicity (3). Non-black race included White, Asian, Native American, Native Hawaiian/Pacific Islander, and other.

**Table 2:** Multivariable regressions for parent language, in-person interpreter use, and length of stay.

Regression	Model 1 ^†^		Model 2 ^†^	
Predictor *	Incidence Rate Ratio (95% Confidence Interval)	*p*-Value	Incidence Rate Ratio (95% Confidence Interval)	*p*-Value
Non-English Primary Language	0.89 (0.77–1.03)	0.118	------	------
Non-English + No Interpreter Use	------	------	0.51 (0.43–0.61)	0.000
Non-English + Low Interpreter Use	------	------	1.26 (1.06–1.51)	0.010
Non-English + High Interpreter Use	------	------	1.41 (0.89–2.22)	0.145
Male Gender	1.17 (1.07–1.29)	0.001	1.20 (1.09–1.31)	0.000
Extreme Low Birth Weight (<1000 g)	2.46 (1.99–3.04)	0.000	2.45 (1.99–3.01)	0.000
Severe Medical Condition (NMI ^‡^ score IV or V)	3.64 (3.12–4.25)	0.000	3.43 (2.94–4.00)	0.000
Black Race	1.00 (0.79–1.26)	0.993	1.00 (0.80–1.25)	0.993
Public Insurance	1.01 (0.89–1.15)	0.853	0.96 (0.85–1.10)	0.576
Married	0.89 (0.79–1.01)	0.074	0.86 (0.76–0.97)	0.017

^†^ Models include non-English primary language and in-person interpreter use, male gender, extreme low birth weight, severe medical condition (neonatal medical index scores IV–V), black race, public insurance, and married status. Hispanic ethnicity was not included in regression models due to high correlation (0.59) with non-English primary language; * Reference groups include English primary language, female gender, birth weight 1001–2500 grams, neonatal medical index scores I–III, non-black race, private insurance, and single/separated/divorced status; ^‡^ Abbreviation: NMI = neonatal medical index.

## Appendix A

**Table A1:** Clinical criteria for scoring the neonatal medical index [21].

Neonatal Medical Index Score	Clinical Criteria
**V**	Meningitis, confirmed or suspected Seizures Periventricular or intraventricular hemorrhage grade III or IV Periventricular leukomalacia Assisted ventilation for >29 days (chronic lung disease, bronchopulmonary dysplasia)
**IV**	Resuscitation needed for apnea or bradycardia while on theophylline Major surgery Patent ductus arteriosus requiring surgical treatment Assisted ventilation for 15–28 days
**III**	Birth weight < 1000 g Periventricular or intraventricular hemorrhage grade I or II Apnea or bradycardia requiring theophylline or related drugs Patent ductus arteriosus treated by indomethacin Hyperbilirubinemia requiring exchange transfusion Assisted ventilation for 3–14 days
**II**	Birth weight > 1000 g Patent ductus arteriosus not requiring drug treatment Oxygen requiring for >1 day Occasional apnea or bradycardia not requiring theophylline or related drugs Assisted ventilation <48 h
**I**	Birth weight > 1000 g Free of respiratory distress Free of major medical complications but may have benign heart murmur or require phototherapy No oxygen required Absence of apnea or bradycardia

## References

[1] Behrman R.E., Butler A.S., IOM (2007). Preterm Birth: Causes, Consequences, and Prevention.

[2] Russell R.B., Green N.S., Steiner C.A., Meikle S., Howse J.L. (2007). Cost of Hospitalization for Preterm and Low Birth Weight Infants in the United States. Pediatrics.

[3] AAP (2008). Hospital Discharge of the High-Risk Neonate. Pediatrics.

[4] Flores G., Olson L., Tomany-korman S.C. (2005). Racial and Ethnic Disparities in Early Childhood Health and Health Care. Pediatrics.

[5] Guerrero A.D., Rodriguez M.A., Flores G. (2011). Disparities in Provider Elicitation of Parents’ Developmental Concerns for US Children. Pediatrics.

[6] Clemans-Cope L., Kenney G. (2007). Low Income Parents’ Reports of Communication Problems with Health Care Providers: Effects of Language and Insurance. Public Health Rep..

[7] Levas M.N., Cowden J.D., Dowd M.D. (2011). Effects of the Limited English Proficiency of Parents on Hospital Length of Stay and Home Health Care Referral for Their Home Health Care–Eligible Children with Infections. Arch. Pediatr. Adolesc. Med..

[8] DHHS (2010). National Standards for Culturally and Linguistically Appropriate Services in Health and Healthcare.

[9] Kuo D.Z., O’Connor K.G., Flores G., Minkovitz C.S. (2007). Pediatricians’ use of language services for families with limited English proficiency. Pediatrics.

[10] Hsieh E. (2015). Not Just “Getting by”: Factors Influencing Providers’ Choice of Interpreters. J. Gen. Intern. Med..

[11] Diamond L.C., Schenker Y., Curry L., Bradley E.H., Fernandez A. (2009). Getting By: Underuse of interpreters by resident physicians. J. Gen. Intern. Med..

[12] Schenker Y., Wang F., Selig S.J., Ng R., Fernandez A. (2007). The Impact of Language Barriers on Documentation of Informed Consent at a Hospital with On-Site Interpreter Services. J. Gen. Intern. Med..

[13] Schenker Y., Pérez-stable E.J., Nickleach D., Karliner L.S. (2011). Patterns of Interpreter Use for Hospitalized Patients with Limited English Proficiency. J. Gen. Intern. Med..

[14] Lee K.C., Winickoff J.P., Kim M.K., Campbell E.G., Betancourt J.R., Park E.R., Maina A.W., Weissman J.S. (2006). Resident Physicians’ Use of Professional and Nonprofessional Interpreters: A National Survey. JAMA.

[15] Ramirez D., Engel K.G., Tang T.S. (2008). Language Interpreter Utilization in the Emergency Department Setting: A Clinical Review. J. Health Care Poor Underserved.

[16] IOM (2007). Crossing The Quality Chasm: A New Health System for the 21st Century.

[17] Bowers J., Cheyne H. (2016). Reducing the length of postnatal hospital stay: Implications for cost and quality of care. BMC Health Serv. Res..

[18] Zhang S., Palazuelos-munoz S., Balsells E.M., Nair H., Chit A., Kyaw M.H. (2016). Cost of hospital management of Clostridium difficile infection in United States—A meta-analysis and modelling study. BMC Infect. Dis..

[19] U.S. Census Bureau: State and County QuickFacts. https://quickfacts.census.gov/qfd/states/06/06085.html.

[20] Lowe H.J., Ferris T.A., Hernandez P.M., Weber S.C. (2009). STRIDE—An Integrated Standards-Based Translational Research Informatics Platform. AMIA Symp..

[21] Korner A.F., Stevenson D.K., Kraemer H.C., Spiker D., Scott D.T., Constantinou J., Dimiceli S. (1993). Prediction of the Development of Low Birth Weight Preterm Infants by a New Neonatal Medical Index. J. Dev. Behav. Pediatr..

[22] Samsom J.F., De Groot L., Cranendonk A., Bezemer D., Lafeber H.N., Fetter W.P.F. (2002). Neuromotor Function and School Performance in 7-Year-Old Children Born as High-Risk Preterm Infants. J. Child Neurol..

[23] López L., Rodriguez F., Huerta D., Soukup J., Hicks L. (2015). Use of Interpreters by Physicians for Hospitalized Limited English Proficient Patients and Its Impact on Patient Outcomes. J. Gen. Intern. Med..

[24] Hendson L., Reis M.D., Nicholas D.B. (2015). Health Care Providers’ Perspectives of Providing Culturally Competent Care in the NICU. J. Obstet. Gynecol. Neonatal Nurs..

[25] Mendoza F.S. (2009). Health Disparities and Children in Immigrant Families: A Research Agenda. Pediatrics.

[26] Callister L.C., Birkhead A. (2002). Acculturation and Perinatal Outcomes in Mexican Immigrant Childbearing Women: An Integrative Review. J. Perinat. Neonatal Nurs..

[27] Bird C., Conrad P., Fremont A., Tinnermans S. (2010). Handbook of Medical Sociology.

[28] Seltz L.B., Zimmer L., Ochoa-Nunez L., Rustici M., Bryant L., Fox D. (2011). Latino Families’ Experiences with Family-Centered Rounds at an Academic Children’s Hospital. Acad. Pediatr..

[29] Miquel-Verges F., Donohue P.K., Boss R.D. (2011). Discharge of Infants from NICU to Latino Families with Limited English Proficiency. J. Immigr. Minor. Health.

[30] Davidson J.E. (2013). Family Presence on Rounds in Neonatal, Pediatric, and Adult Intensive Care Units. Ann. Am. Thorac. Soc..

[31] Lindholm M., Hargraves J.L., Ferguson W.J., Reed G. (2012). Professional Language Interpretation and Inpatient Length of Stay and Readmission Rates. J. Gen. Intern. Med..

[32] Karliner L.S., Napoles-Springer A.M., Schillinger D., Bibbins-Domingo K., Pérez-Stable E.J. (2008). Identification of Limited English Proficient Patients in Clinical Care. J. Gen. Intern. Med..

[33] Burnham N., Feeley N., Sherrard K. (2013). Parents’ Perceptions Regarding Readiness for Their Infant’s Discharge from the NICU. Neonatal Netw. J. Neonatal Nurs..

[34] Eneriz-wiemer M., Liu S., Chu M.C.Y., Uribe-Leitz T., Rajani K., Sankar M., Robbins S.L., Lee H.C., Woodard C., Wang C.J. (2018). Parents’ Knowledge and Education of Retinopathy of Prematurity in Four California Neonatal Intensive Care Units. Am. J. Ophthalmol..

